# Additional Remarks on an Obstetrical Case

**Published:** 1868-10-01

**Authors:** W. Anderson

**Affiliations:** Leroy, Ill.


### ADDITIONAL REMARKS ON AN OBSTETRICAL CASE.

BY W. ANDERSON, M.D., LEROY, ILL.

EDITORS JOURNAL: I do not purpose to go into a review of the lengthy article by Dr. Miller and other subsequent contributors, in regard to my report of "instrumental case of delivery," but as my report was very brief, I wish to simply add a few more statements in order to render the case more comprehensible to the dull understandings of the "sage practitioners" who have condescended to throw so much light on this department of midwifery.

I may now add (which for the sake of brevity I neglected to do at first) that there were three physicians present, all of whom had had extensive experience in obstetrics, and two of whom were graduates, the third being Dr. Leal, of Mt. Pleasant, a young man of a very fine practice and repute. We did not differ as to method of procedure. *Chloroform* was talked of, but there was none present, and it was five miles to town, and a very cold night. I stated that we would not have used *Chloroform* had it been at hand — the child dead or alive. That remark was inadvertent. We might have tried it had the child been alive, though I think even then it would have been unavailable. But the child was dead, and its use under the circumstances was not necessary, and we all thought unadvisable, as a recent death from anæsthesia in Bloomington had created a great antipathy against its use. The arm had protruded as far as the elbow, was black and pulseless, and all the signs plainly indicated the death of the fœtus. The family physician had been present twelve hours. Why he waited so long is not for me to say. He was not called till quite late in labor, and the hand had been down for two or three days. I believe the child might have been saved at the right time, and it had been dead but a short time when I arrived. I was called in on account of my instruments. The attending physician had been waiting simply for "something to turn up." He is an "irregular," in the highest sense of that term, but the consulting physicians are still willing to bear all the responsibility for all the proceedings after they arrived. I think the above statements are as satisfactory as a long discussion, and require less space. I would not have alluded to the family physician were it likely to hurt his feelings by his ever reading it, and the statements render it unnecessary to reply to the numerous communications on the subject in detail, even if the slang in some of them *did* not place them beneath my notice.

